# The Conjunction and Disjunction Fallacies: Explanations of the Linda Problem by the Equate-to-Differentiate Model

**DOI:** 10.1007/s12124-015-9314-6

**Published:** 2015-06-17

**Authors:** Yong Lu

**Affiliations:** Faculty of Theology, Cardinal Stefan Wyszyński University in Warsaw, ul. Dewajtis 5, Warsaw, 01-815 Poland

**Keywords:** Conjunction fallacy, Disjunction fallacy, Equate-to-differentiate model, Pragmatic heuristic

## Abstract

We propose the use of the equate-to-differentiate model (Li, S. (2004), Equate-to-differentiate approach, *Central European Journal of Operations Research*, *12*) to explain the occurrence of both the conjunction and disjunction fallacies. To test this model, we asked participants to judge the likelihood of two multi-statements and their four constituents in two modified versions of the Linda problem in two experiments. The overall results underpin this pragmatic model’s inference and also reveal that (1) single conjunction and disjunction fallacies are most prevalent, (2) the incidence of the conjunction fallacy is proportional to the distance between the constituent probabilities, and (3) some participants misinterpreted *A* ∧ *B* either as ¬ *A* ∧ *B* or *A* ∨ *B*. The findings were generally consistent with the configural weighted average model (Nilsson, H., Winman, A., Juslin, P., & Hansson, G. (2009), Linda is not a bearded lady, *Journal of Experimental Psychology*: *General*, *138*) and the potential surprise conceptual framework (Fisk, J. E. (2002), Judgments under uncertainty, *British Journal of Psychology*, *93*).

## Introduction

Traditional assumptions about rationality presume that when people deduce, their judgment should abide by Bayes’ Rule (Morris 1974, 1977) and should not be affected by semantical descriptions. However, since Simon (1957) proposed the idea of *bounded rationality*, a significant amount of experimental and field evidence suggests that people sometimes do not perform perfectly due to inner or outer factor restrictions such as cognitive limitations, logical errors, misapprehensive implication, pressured time allocation, or varying contents. In particular, studies based on probability judgment suggest that people often show biases (e.g., base-rate neglect, conjunction fallacy, disjunction fallacy, hindsight bias, overconfidence, sample-size neglect) in the probabilistic (Bayesian) inference tasks and therefore violate some fundamental key properties of classical probability theory. We note that the conjunction and disjunction fallacies are two related phenomena that have been well-researched in the past 30 years and become two necessary components in bias and fallacy studies.

### Conjunction and Disjunction Fallacies

The *conjunction fallacy* explores how individuals commonly violate a basic probability rule by estimating probability of conjunction of two statements to be more probable than the probability they assign to at least one of its constituent statements. Tversky and Kahneman (1983) first proposed the conjunction fallacy. In their seminal study, they presented participants with an afterwards well-known probability judgment scenario named the Linda task. A hypothetical woman named Linda as well as a personality sketch on some of her characteristic and activities functioned as the target *E* on which they later asked the participants to make judgment about Linda.

(*E*) *Linda is 31 years old*, *single*, *outspoken*, *and very bright. She majored in philosophy. As a student*, *she was deeply concerned with issues of discrimination and social justice*, *and also participated in antinuclear demonstrations*.

After reading the description of that target *E*, they requested the participants to estimate the probability of a number of statements that were true referring to *E*. Three statements are included as follows:

(T) Linda is a bank teller.
(F) Linda is active in the feminist movement.
(T ∧ F) Linda is a bank teller and is active in the feminist movement.

The participants should estimate the individual statement *T* as more likely than the conjunction *T* ∧ *F* since it is impossible for Linda to be a feminist bank teller without also being a bank teller. However, a significant amount of later studies found that the majority of respondents commit single conjunction fallacy and even double conjunction fallacy (e.g., Abelson, et al. 1987; Fantino, et al. 1997). The single conjunction fallacy means that respondents judge the conjunctive estimate being higher than one of the constituent and being lower than the other constituent. The double conjunction fallacy means that respondents judge the conjunctive estimate being higher than both of the constituents. The observed high error rate is most extraordinary and was even up to 87 % in Tversky and Kahneman (1983). Strikingly, even in a distinct and succinct Linda problem in which only three statements *T*, *F*, and *T* ∧ *F* were presented to participants, it was still found in Tversky and Kahneman (1983) that 85 % of the participants rated *T* ∧ *F* as more probable than *T*. A number of studies found that a range of boundary factors, e.g., feedback (Charness et al. 2010), hint (Brachinger and Monney 2003), individual differences (Feeney et al., 2007; Fisk 2005; Morsanyi et al. 2010; Stanovich and West, 1998), economic incentives (Charness et al. 2010; Zizzo et al. 2000), response formats (Ellen 2000; Hertwig and Gigerenzer 1999; Von Sydow 2011; Wedell and Moro 2008), and source reliability (Bovens and Hartmann 2003), influence the incidence of the conjunction fallacy. The accumulated evidence of experimentation from 1980s has suggested that the violation is highly robust to variations in response modes and is very easy to replicate in a variety of contexts.

In a similar violation of probability theory, the *disjunction fallacy* shows that people estimate a disjunctive statement to be less probable than at least one of its component statements (e.g., Bar-Hillel and Neter 1993; Carlson and Yates 1989). When people judge that the disjunctive estimate is higher than one of the constituent and is lower than the other constituent, they commit the single disjunction fallacy. When people judge that the disjunctive estimate is lower than both of the constituents, they commit the double disjunction fallacy. A handful of studies focused on the disjunction fallacy and found relevant evidence on its happening. For example, Young et al. (2007) found that adding a stigmatized vector (e.g., unprotected sex) to a list of otherwise innocuous possible vectors causes reduced estimated likelihood, in violation of disjunction probability rule. Lambdin and Burdsal (2007) rectified the claims of Kuhberber et al. (2001) that people do not violate the sure-thing principle in repeated gambles and further suggested that people do regularly violate the sure-thing principle in two-step gambles, providing evidence for the reality of disjunction effects. Li et al. (2012) confirmed the reason-based account on explanation of the disjunction fallacy. Since manifesting a similar fallacious procedure comparing with the conjunction fallacy, the disjunction fallacy was not studied too much, and the relevant researches still mainly focused on the conjunction fallacy.

There are four critical explanations as to why many manifestations of this fallacious behavior on conjunctive probability judgment might happen. The initial explanation ascribes the fallacy as that people rely on some psychological relations such as the representativeness and availability theory models which arouse the conjunction fallacy (e.g., Bar-Hillel and Neter 1993; Brachinger 2005; Tversky and Kahneman 1983). Concretely, Tversky and Kahneman (1983) considered that different problem types would induce people to apply different judgment heuristics. When people are presented a problem with dramatic meaning, they are more deeply appealed by its content. Therefore, their attentions no longer focus on using principles of probability to judge it. However, these heuristics have been criticized heavily by Gigerenzer (1996) as being far “too vague to count as explanations” (p. 593) and “lack theoretical specification” (p. 594). Also Gavanski and Roskos-Ewoldsen (1991) found representativeness involved insofar as it only influences the probabilities of component statements. Therefore, many accumulated evidence (others see Wolford et al. 1990; Yates and Carlson 1986, etc.) have shown opposite opinions against the representativeness and availability heuristics’ interpretation on the conjunction fallacy.

A second major interpretation postulates that the conjunction fallacy is unquestionably considered a probabilistic error (e.g., Bar-Hillel and Neter 1993; Costello 2009; Tversky and Kahneman 1983) or linguistic misunderstanding (e.g., Hartmann and Meijs 2012; Hertwig et al. 2008; Macdonald and Gilhooly 1990; Politzer and Noveck 1991; Wolford et al. 1990). For instance, Costello (2009) proposed that participants represent the conjunction as an effect of random error in the judgment process. It is quite obvious that when people mistake *A* ∧ *B* into *A* ∨ *B*, the probability of the “conjunctive” statement is larger than its components’ probabilities. Wolford et al. (1990) (see also Wolford 1991) proposed that participants misunderstand conditional probabilities of *T*∣*E*, *F*∣*E* and (*T* ∧ *F*)∣*E* to *E*∣*T*, *E*∣*F* and *E*∣(*T* ∧ *F*) respectively. Politzer and Noveck (1991) argued that the task demands are likely to compel participants to misinterpret a base statement (e.g., *T*) as the conjunction of the base statement and the complement of an added statement (e.g., *T* ∧ ¬*F* [Linda is a bank teller and she is not a feminist]). Therefore, when assuming that *T* and *F* are independent and that participants’ probability estimate on *F* is larger than 0.5, the probability of *T* ∧ *F* is larger than the probability of *T* ∧ ¬*F*. However, Moro (2009) found that the rate of violations of the conjunction rule remains prevalent by explicitly including the statement *T* ∧ ¬*F* along with *T* as well as *T* ∧ *F* in the judgment task (also see Tentori et al. 2004; Wedell and Moro 2008), which questions the theoretical tenability of Politzer and Noveck’s (1991) argument. Furthermore, Tentori and Crupi (2012) obtained results overtly contradictory to the claims of Hertwig et al. (2008) that unintended misinterpretations of the logical connective “*and*” emerged from “reasonable pragmatic inferences” (p. 752) may account for behaviour of the conjunction fallacy. It is theoretically argued by Tentori and Crupi (2012) that, firstly, it is uncontroversial that different interpretations of the word “*and*” across different sentences do not imply anything about the word’s ambiguity within a given sentence. Second and majorly, even when the word “*and*” is not exhausted by the logical operator ∧, its interpretation often legitimizes application of the conjunction rule all the same. In short, the linguistic misunderstanding explanation attributes the conjunction and disjunction fallacies to significantly different meanings of the questions and of participants’ interpretations.

On the other hand, some critical explanations that have been proposed based on Bayesian solutions suggested that the conjunction fallacy might not be fallacious in certain circumstances (among others Bovens and Hartmann 2003; Busemeyer et al. 2011; Tentori et al. 2013). For instance, Bovens and Hartmann (2003) explicitly argued from source reliability perspective that the conjunction fallacy can be accounted for in a Bayesian framework given prior beliefs in the likelihood of Linda being a feminist given her background description. They argue that participants who believe *T* ∧ *F* more than *T* are rational if and only if: Δ*Prob* = *Prob* (*T*, *F*|Rep*T*, Rep*F*) - *Prob* (*T*|Rep*T*) > 0, where Rep*T* denotes a report of *T* by the participants’ certain witness scenario. If so, the participants would, in a Bayesian perspective, not be committing a reasoning fallacy when responding that *T* ∧ *F* is more likely than *T*. Besides, Busemeyer et al. (2011) proposed that in accordance with a generalization of Bayesian probability theory, quantum probability model can explain the conjunction fallacy, though Tentori and Crupi (2013) argued against their approach’s explanation. On the other hand, Tentori et al. (2013) put forth new empirical findings as defined by contemporary Bayesian theory of argument that the conjunction fallacy depends on the added conjunct (e.g., *F*) being perceived as *inductively confirmed* rather than some of competing explanations’, e.g., the averaging hypothesis (Fantino et al. 1997), the random error model (Costello 2009), proposals that the conjunction fallacy rates would rise as the *posterior probability* of the added conjunct does. Then Tentori et al. (2013) argued that their results cannot be explained by those prevalent judgment models and therefore provided new evidence for the role of inductive confirmation as a major determinant of the conjunction fallacy.

The fourth perspective assumes that the conjunction fallacy is aroused by incorrectly using certain integrate computing models, i.e., compensatory strategies, or heuristic models, i.e., non-compensatory strategies (see Betsch and Fiedler 1999 and Gavanski and Roskos-Ewoldsen 1991 for a discussion). These integrate computing models include proposals as diverse as averaging rule hypotheses (Fantino et al. 1997), configural weighted average model (Juslin et al. 2009; Nilsson et al. 2009), conjunction coefficient model (Abelson et al. 1987), fuzzy logical model of perception (Massaro 1994), random error model (Costello 2009), and signed sum model (Yates and Carlson 1986). For instance, Fantino et al. (1997) proposed that people non-normatively average the likelihood of the two components in arriving at a judgment of the likelihood of the conjunction. On the other hand, these heuristic models include proposals such as potential surprise hypothesis (Fisk 2002) and the reasoning bias hypothesis (Moro 2009). Although incorrectness comparing with the probability theory and even several of the integrative computing models are very unlikely from a psychological point of view, both the integrative and heuristic models explain when and why fallacious behaviors appear or disappear.

The question of whether people rely on psychological relations, Bayesian rules (no matter whether Bayesian probability theory is believed in the correct way or other kind of understandings), the integrative models, or the heuristic models in their judgment is still in dispute (e.g., Denes-Raj and Epstein 1994; Kemmelmeier 2009). Still there are some evidences that support the psychological relations interpretation, such as the representativeness theory (Brachinger 2005; Wells 1985). At the same time, the formal Bayesian frameworks (notably classical probability theory) are still prosperous (e.g., recently Crupi et al. 2008; Hartmann and Meijs 2012; Shogenji 2012; Von Sydow 2011) but are also questioned from the growing studies on bounded rationality that realizes the limitations of the human mind and the structure within which the mind operates, even such as in some Bayesian frameworks on the conjunction fallacy (e.g., Brachinger 2005; Franco 2007). On the other hand, the integrative models’ interpretation has been developed as the substitutive rules to compensate people’s incorrect use of Bayes’ Rule. Those integrative models, e.g., the weighting and summing calculation process (Nilsson et al. 2009), assume as usual as the expectation rule that people should be competent for the needed quantitative calculation. By contrast, the heuristic models’ interpretation sheds light on people’s non-compensatory strategies and proposes a bounded rationality perspective on the phenomena. Furthermore, the heuristic models highlight people’s fundamental and underlying cognitive processes more closely than the integrative models that emphasis on outcome prediction or goodness-of-fitting. Especially, recent studies, e.g., Birnbaum and LaCroix 2008; Brandstatter et al. 2006; Wang and Li 2012, have accumulated some evidence that supports for the heuristic models (for the integrative vs. heuristic models’ debate, see Gigerenzer and Selten 2001 for a discussion). Some tendencies (e.g., Mosconi and Macchi 2001) have also been gained to employ these heuristic conceptual approaches to veritably model human judgment illusions under uncertainty, such as the conjunction fallacy. The attempt to employ the heuristic models for modeling cognition has enabled the introduction of several new concepts in psychology, such as simple heuristics, ecological or pragmatic rationality, and bounded rationality.

Now that there are different theoretical explanations of the conjunction and disjunction fallacies, it appears obviously doubtful that there is a univocal mechanism that fully attributes to all the phenomena. At the same time, there may well be other approaches that reflect underlying mental processes of the phenomena. Therefore, in the next part, we propose the equate-to-differentiate model (Li 2004), a heuristic model, to explain the phenomena.

### Assumption of the Equate-to-Differentiate Model’s Explanation of the Conjunction and Disjunction Fallacies

The equate-to-differentiate model (Li 2004) assumes that when people make judgments or choices among a few propositional statements (e.g., concerning occupations or personality dispositions), people implement such a judgmental process by filtering one or several less distinct dimension(s) of each statement. Furthermore, the model assumes that people base their judgments of the relative likelihoods of the conjunctive/disjunctive and single statements on the values derived from the most distinct dimension of each statement (while neglecting other less distinct dimension(s)). The most distinct dimensions of a statement *A* and another statement *B*, for example, are the ones that exist at least one *j* such that | $\left|{U}_{A_j}\left({x}_j\right)-{U}_{B_j}\left({x}_j\right)\right|=\left|{U}_{A_{j0}}\left({x}_j\right)-{U}_{B_{j0}}\left({x}_j\right)\right|$ having subjectively treated all | $\left|{U}_{A_j}\left({x}_j\right)-{U}_{B_j}\left({x}_j\right)\right|<\left|{U}_{A_{j0}}\left({x}_j\right)-{U}_{B_{j0}}\left({x}_j\right)\right|$ as ${U}_{A_j}\left({x}_j\right)$ = ${U}_{B_j}\left({x}_j\right)$, where *x*_*j*_ (*j* = 1,…,*m*) is the objective value of each statement on dimension *j* and | $\left|{U}_{A_{j0}}\left({x}_j\right)\right|-\left|{U}_{B_{j0}}\left({x}_j\right)\right|$ = max {${\displaystyle {\sum}_{j=1}^m\Big|{U}_{A_j}\left({x}_j\right)}-{U}_{B_j}\left({x}_j\right)\Big|$}. On the other hand, the less distinct dimensions of *A* and *B* are the rest ones besides the most distinct dimensions. One statement with a larger outcome of its most distinct dimension is preferred to another statement with a less outcome of its most distinct dimension.

**Fig. 1:**
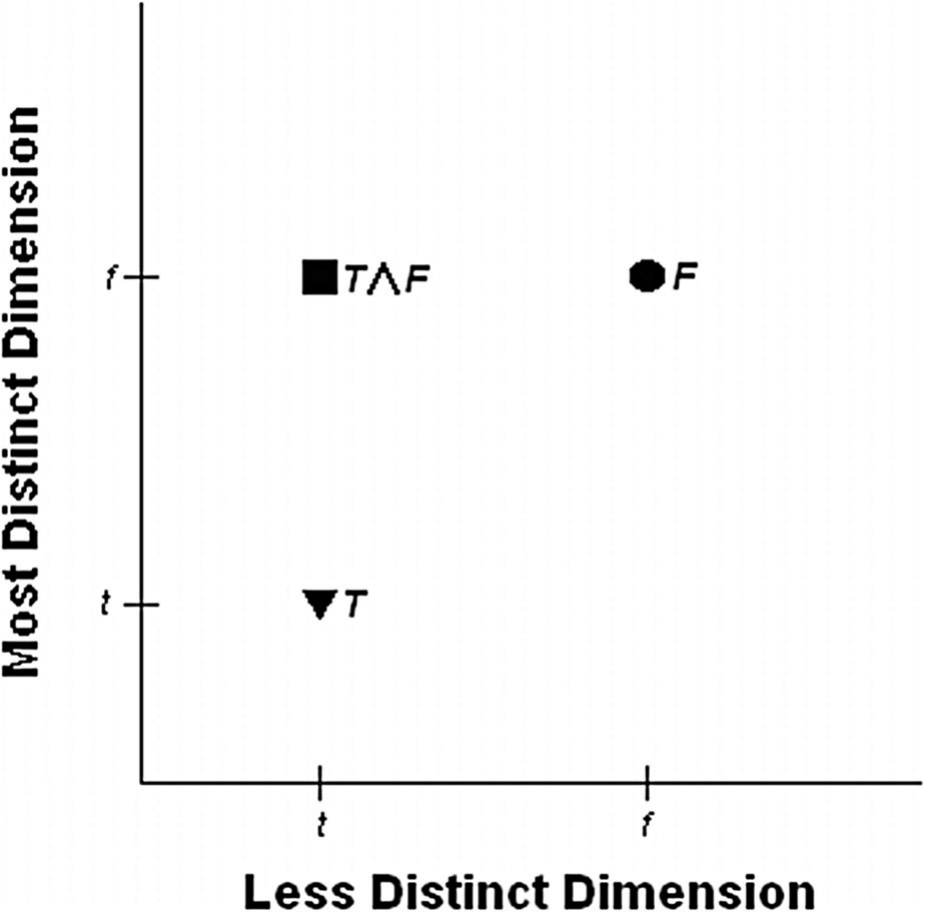
The equate-to-differentiate model’s interpretation of the Linda problem (when a decision maker judges *f* > *t*). *Note* according to the equate-to-differentiate model (Li 2004), the decision maker decomposes the statements *T*, *F* and *T* ∧ *F* into the less distinct dimension (the horizontal axis) and the most distinct dimension (the vertical axis). Vector *t* on the horizontal axis, and the vertical axis, denotes to the objective value of the statements *T* and *T* ∧ *F* on the less distinct dimension, and the most distinct dimension, respectively. Vector *f* on the horizontal axis, and the vertical axis, denotes to the objective value of the statements *F* and *T* ∧ *F* on the less distinct dimension, and the most distinct dimension, respectively. The outcomes *t* or *f* itself can be seen as either the less distinct objective value or the most distinct objective value

As concerns the standard conjunctive Linda problem, the model presumes that a decision maker uses only the subjective marginal probability about *T* ∧ *F* and uses the information of one of the two involved dimensions of *T* ∧ *F* ($\mathbb{T}$ vs. $\mathbb{F}$) in the first place (see Fig. 1 for representing the model’s interpretation of the standard conjunctive Linda problem). Suppose the decision maker judges *f* ≥ *t*, where *t* and *f* are the probabilities of *T* and *F* and can also be seen as the objective values of the dimensions $\mathbb{T}$ and $\mathbb{F}$, respectively. In the case of comparing *T* with *T* ∧ *F*, the less distinct dimension ($\mathbb{T}$) is equally present in *T* and in *T* ∧ *F* and therefore yields equal outcomes. Then the decision maker restricts the situation to only another two dimensions, the most distinct dimensions $\mathbb{T}$ of *T* and $\mathbb{F}$ of *T* ∧ *F*, respectively. Only in this case, in a second model step the most distinct dimensions are consulted as well: $\mathbb{T}$ is hence compared to $\mathbb{F}$, then the decision maker yields (*T* ∧ *F*) ≽_L_ *T*.^1^ Similarly, in the case of comparing *F* with *T* ∧ *F*, the less distinct dimension ($\mathbb{F}$) is equally present in *F* and in *T* ∧ *F* and therefore yields equal outcomes. Only in this case, in a second model step the most distinct dimensions are consulted as well: $\mathbb{F}$ of *F* is hence compared to $\mathbb{T}$ of *T* ∧ *F*, then the decision maker yields *F* ≽ _L_*T* ∧ *F*.

In sum, the model predicts that the decision maker would err in the conjunctive Linda problem by judging *F* ≽_L_ (*T* ∧ *F*) ≽_L_ *T* when he judges *f* ≥ *t*. Similarly, the prediction for the disjunctions is identical to those for the conjunctions. In the standard disjunctive Linda problem, the model predicts that the decision maker would commit disjunction fallacy by judging *F* ≽_L_ (*T* ∨ *F*) ≽_L_ *T* when he judges *f* ≥ *t*. The theoretical hypothesis of interpreting the conjunction fallacy by the model can also address comparisons between several conjunctions (or disjunctions) or more complex conjunctions (or disjunctions), but this more complex situation will not be discussed in the current paper.

### The Present Study

The legitimate and primary goal of the present study is to add a new equate-to-differentiate model’s (Li 2004) explanation on the documented catalogue of the conjunction and disjunction fallacies. In two experiments, the judged likelihood of four components constituted two combinatorial statements, and they were varied in scenario of the most-studied Linda problem to test the model’s explanations.

Second, taking into account a number of existing combination rules put forward so far to explain the biases, the equate-to-differentiate model was compared with the configural weighted average model (CWA, Nilsson et al. 2009), potential surprise model (Fisk 2002) and signed sum model (Yates and Carlson 1986) to see which patterns in data that supported the equate-to-differentiate model but that cannot be accounted for by the candidate models. It should be noted that the data from Experiment 1 to 2 potentially could be explained by the three candidate models and only how well the data fit the equate-to-differentiate model was discussed in the present paper.

Third, in Experiment 2 a Venn diagram task was designed to investigate participants’ understood meaning of the conjunctive connection word “and”.

In two experiments, we tested two predictions. Since the equate-to-differentiate model’s explanation indicates that the larger constituent would be judged more probable than the compound combination and the compound combination would be judged more probable than the smaller constituent, we hypothesized that first, the single conjunction (disjunction) fallacy should be more exceptionally frequent than both the zero conjunction (disjunction) fallacy and the double conjunction (disjunction) fallacy.

In any conjunction fallacy problem, the key statements have the following form, independent of whether they are likely or unlikely: (a) *A*; (b) *A* ∧ *B*. Suppose the statement *A* is called the *base statement* because it appears by itself in the first statement and constitutes the basis for the construction of the second statement where the statement *B* is added. The statement *B* is called the *added statement*. As the distance between the likelihoods of the base statement and added statement increases, the difference between the dominated dimensions also increases. Thus, if suppose people use the equate-to-differentiate model (Li 2004) to estimate probabilities, they are necessarily going to commit the conjunction fallacy since there is a distinct discrepancy between the dominated dimensions of *A* and *A* ∧ *B*. Notice that if the base statement and the added statement were all likely or unlikely, people rarely commit the conjunction fallacy even if they are using the equate-to-differentiate model, because there is no distinct dominated dimension in feminist bank teller than in feminist alone. Therefore, we hypothesized that second, conjunction fallacy should occur more frequently in the likelihood type of Unlikely ∧ Likely than the likelihood types of Unlikely ∧ Unlikely and Likely ∧ Likely. In fact, variations in the likelihoods of component statements have also been demonstrated to induce different conjunction fallacy (e.g., Fantino et al. 1997; Nilsson et al. 2009; Tversky and Kahneman 1983; Yates and Carlson 1986).

The first prediction was tested in Experiments 1 and 2, and the second prediction was tested in Experiment 2.

## Experiment 1

Experiment 1 had two goals. The first was to investigate whether participants’ probability estimates for disjunctive statements and their constituents are in accordance with the predictions by the equate-to-differentiate model (Li 2004). The second was to extend and examine the previous findings (Fisk 2002 and Nilsson et al. 2009).

### Method

#### Participants

Forty-five Chinese undergraduate students specializing in Engineering Management at Tianjin University volunteered to take part in the study. Four participants did not complete the questionnaires (by omitting one or several probabilities of single or disjunctive statements) and therefore were discarded from subsequent data analyses.

#### Design, Procedure, and Materials

A within-subjects design was used.

The participants were assembled in one class. As the study presented no more than minimal risk of harm to the participants and involved no procedures for which written consent is normally required outside of the study context, the Ethics Committee specially approved the study as well as verbal informed consent being provided to the participants in lieu of signed informed consent. The experimenter (myself) read out loud the verbal informed consent at the outset. All of the participants consented to participate in the experiment and then received a booklet containing a task instruction, a narrative description of Linda borrowed from the New Linda Unknown Outcomes Scenario (Wolford et al. 1990), and a target task presented in tabular form. Those participants were asked to assign probabilities to two disjunctions, their component statements (see Table 1 for the statements), and another irrelevant single statement. The participants were invited to use a number in the whole range 0 to 1 (including 0 and 1) either by decimal, fractional, or percent estimates as a reply to each statement. It was mentioned in the instructions that when they forecast that a statement is *unlikely* to happen, they should determine its probability within the range of 0 to 0.50, and when they forecast that a statement is *likely* to happen, they should determine its probability within the range of 0.5 to 1, where 0 means minimal probability and 1 means maximal probability. Questions were answered individually at the participants’ seats.

### Results and Discussion

#### Probabilities

Table 1 shows the means and median probability estimates for the four single and two disjunctive statements, with standard errors (95 % confidence interval) in parentheses. Results show that the average probability estimates for both *T* ∨ *F* and *Y* ∨ *P* are not significantly smaller than the average probability estimates for the likely target items with an alpha level of 5 %. The single statement *T* received by far the lowest probability estimates.

**Table 1:** Means and median probability estimates in Experiment 1

	Probability estimates	
Items^a^	Mean (%)^b^	Median (%)
Linda will be a teacher in elementary school. (P)	29.3 (3.7)	20
Linda will be active in the feminist movement. (F)	71.3 (2.9)	80
Linda will be a bank teller. (T)	22.5 (3.4)	10
Linda will take Yoga classes. (Y)	42.5 (4.4)	50
Linda will be a bank teller or will be active in the feminist movement. (T ∨ F)	61.5 (4.0)	65
Linda will take Yoga classes or will be a teacher in elementary school. (Y ∨ P)	46.3 (3.9)	50

^a^In the version given to the participants, the labels *P*, *F*, *T*, *Y*, *T* ∨ *F* and *Y* ∨ *P* were omitted

^b^Standard errors with 95 % confidence intervals are in parentheses. Data indicates no significant difference on the disjunction statements, respectively relative to the likely target items *F* and *Y* (*p* < .05)

#### Components and Disjunction Fallacy

Respectively 17 (41.5 %) and 18 (43.9 %) of the 41 participants estimated that the probability of the component *T* or *F* and that the probability of the component *Y* or *P* were higher than the probabilities of their corresponding disjunctions *T* ∨ *F* and *Y* ∨ *P*. And respectively 4 (9.8 %) and 5 (12.2 %) of the 41 participants thought that both the constituents *T* and *F* and that both the constituents *Y* and *P* were more probable than their corresponding disjunctions *T* ∨ *F* and *Y* ∨ *P*. Across all the 41 participants’ probability estimates for the four constituents, respectively 20 (48.8 %) and 18 (43.9 %) of their estimates were consistent with the disjunctive rule when they judged the disjunctive statements *T* ∨ *F*, *Y* ∨ *P*, and their corresponding components; respectively 21 (51.3 %) and 23 (56.1 %) of their estimates committed the disjunction fallacy when they judged the disjunctive statements *T* ∨ *F*, *Y* ∨ *P*, and their corresponding components. Chi-squared tests revealed that the single disjunction fallacy was higher than the double disjunction fallacy (*χ*^2^ = 15.364, *p* < .001), but that the single disjunction fallacy was not different from the zero disjunction fallacy (*χ*^2^ = .123, *p* = .725).

According to the CWA (Nilsson et al. 2009), P (*A* ∨ *B*) = *β*P (*A*) + (1-*β*) P (*B*) (where P (*A*) < P (*B*) and the coefficient *β* is the relative weight of the two components, 0 < *β* < .5). The CWA predicts that both P (*A*) and P (*B*) should influence P (*A* ∨ *B*), and that P (*A*) should attract less weight than P (*B*) if regression analyses are run with smaller and larger constituent probabilities as factors and disjunctive probabilities as dependent variables (also see Fisk 2002).

Multiple regression and partial correlation analyses (see Table 2) were respectively run for the participants’ probability estimates of the disjunctive statements *T* ∨ *F*, *Y* ∨ *P*, the total disjunctive statements of *T* ∨ *F* and *Y* ∨ *P*, and their corresponding smaller and larger components. The regression equation is $\overset{\frown }{P\left(A\vee B\right)}$ = *c*_1_ + *β*_Large_ × P (*B*) + *β*_Small_ × P (*A*), where P (*A*) < P (*B*) and *c*_1_ is a constant term. Results of the regression and partial correlation analyses were broadly consistent with the assertion of both the CWA (Nilsson et al. 2009) and the potential surprise (Fisk 2002) that the probability of the larger component is invariably the more significant one in determining the probability of its disjunctive statement.

**Table 2:** Probability estimates of the larger and smaller component in determining the value assigned to the disjunctive statements in Experiment 1: regression and partial correlation analyses results

	*R* ^2^	Standard coefficient – *β*		Partial correlation coefficient – *β*		*N*
Statement		L*arge*	S*mall*	L*arge*	S*mall*	
T ∨ F	.205	.346^*^	.245	.358^*^	.262	41
Y ∨ P	.506	.533^***^	.286^*^	.558^***^	.340^*^	41
Total	.377	.497^***^	.248^*^	.518^***^	.289^*^	82

^*^ *p* < .05

^***^ *p* < .001

#### Quantitative predictions

To explore to what extent the equate-to-differentiate model provides a good quantitative account of the data, the participants’ probability estimates on the two disjunctive statements and their four components were studied. For a disjunctive statement, the outcomes of the less or most distinct dimension can be regarded as the probabilities of the corresponding component statements constituting to the disjunctive statement. For example, the probability of *T* can be regarded as the outcome of the less distinct dimension $\mathbb{T}$ of the disjunctive statement *T* ∨ *F*. The relative discrepancies between each pair of the outcomes of the disjunctive statements are assumed to determine whether the corresponding two dimensions are the less or most distinct dimensions. For example, since 16 of the 41 participants judged the relative discrepancy between the probabilities of *F* and *P* as the most distinct one, the dimensions $\mathbb{F}$ and ℙ were assumed as the most distinct dimensions. Then the less distinct dimensions $\mathbb{T}$ and $\mathbb{Y}$ were filtered, so the preference between *T* ∨ *F* and *Y* ∨ *P* was determined by the preference between *F* and *P*. As they judged *F* ≻ _L_*P* and (*T* ∨ *F*) ≻ _L_ (*Y* ∨ *P*), their responses were classified as in accordance with the criterion of the equate-to-differentiate model.

In sum, 34 (82.9 %) of the 41 participants’ responses were in accordance with the predictions of the equate-to-differentiate model. Thus, overall, the results confirmed the expectations set out above and were consistent with the model’s account of the disjunctive probability judgment.

## Experiment 2

Experiment 2 had three goals. The first was to test whether participants’ probability estimates for conjunctive statements and their constituents are in accordance with the equate-to-differentiate model’s predictions. The second was to extend and examine the previous findings (Fisk 2002; Nilsson et al. 2009; Yates and Carlson 1986). The third was to explore participants’ semantic interpretations on conjunctive word “*and*” in a Venn diagram task.

### Method

#### Participants

Two hundred and fifty Chinese bachelor students of first, second, and third-year grades specializing in Chemistry Engineering at University of Shanghai for Science and Technology volunteered to take part in the study. The ages of the participants were between 17 and 22 years old, and the mean age was 19.8. The female percentage of the participants was 54 %.

#### Design

A within-subjects design was used. We investigated in four variations (groups) of the Linda task assessing various affirmative events and various conjunctions (see Table 3 for the conjunctive statements and their constituents used in the stimulus through Group 1 to 4). Therefore, the assessment in the likelihood types of low-low, high-low, high-high marginal probabilities can be useful to test the second prediction.

**Table 3:** Means and median probability estimates in Experiment 2

	Probability estimates	
Items^a^	Mean (%)^b^	Median (%)
Group 1 (*N* = 104 ^c^):		
Linda is a bank teller. (T)	13.3 (1.2)	10
Linda is active in the feminist movement. (F)	78.1 (1.8)	80
Linda takes Yoga classes. (Y)	42.0 (2.2)	50
Linda is a teacher in elementary school. (P)	17.7 (1.5)	15
Linda is a bank teller and is active in the feminist movement. (T ∧ F)	**30.2** (**2.5**)	30
Linda takes Yoga classes and is a teacher in elementary school. (Y ∧ P)	**22.3** (**2.1**)	15.5
T ∧ F interpreted as an intersection.^c^ (*n* = 54)	**27.9** (**2.9**)	30
T ∧ F interpreted as two separations.^c^ (*n* = 9)	12.3 (4.3)	10
T ∧ F interpreted as neither an intersection nor two separations.^c^ (*n* = 40)	**41.7** (**4.5**)	40
Group 2 (*N* = 37):		
Linda is a bank teller. (T)	32.2 (4.2)	30
Linda is active in the feminist movement. (F)	67.5 (3.8)	70
Linda is an executive. (D)	39.9 (4.3)	40
Linda subscribes to a popular liberal magazine. (M)	72.0 (3.8)	80
Linda is a bank teller and is active in the feminist movement. (T ∧ F)	**38.6** (**4.2**)	40
Linda is an executive and subscribes to a popular liberal magazine. (D ∧ M)	**49.8** (**4.1**)	50
T ∧ F interpreted as an intersection.^c^ (*n* = 23)	35.7 (6.1)	20
T ∧ F interpreted as two separations.^c^ (*n* = 6)	31.7 (7.9)	35
T ∧ F interpreted as neither an intersection nor two separations.^c^ (*n* = 7)	21.4 (5.5)	10
Group 3 (*N* = 41):		
Linda is an avid reader. (R)	72.1 (2,8)	80
Linda is active in the feminist movement. (F)	72.2 (2.9)	80
Linda is an executive. (D)	40.0 (3.0)	40
Linda subscribes to a popular liberal magazine. (M)	68.6 (3.5)	75
Linda is an avid reader and is active in the feminist movement. (R ∧ F)	**66.9** (**3.1**)	70
Linda is an executive and subscribes to a popular liberal magazine. (D ∧ M)	**44.5** (**3.3**)	50
R ∧ F interpreted as an intersection.^c^ (*n* = 24)	70.4 (3.9)	80
R ∧ F interpreted as two separations.^c^ (*n* = 6)	73.3 (8.4)	75
R ∧ F interpreted as neither an intersection nor two separations.^c^ (*n* = 10)	77.5 (3.9)	80
Group 4 (*N* = 42):		
Linda is a bank teller. (T)	24.3 (3.2)	20
Linda is very shy. (S)	11.7 (2.3)	7
Linda is a teacher in elementary school. (P)	43.7 (4.5)	50
Linda is active in crafts like needlepoint. (C)	31.2 (3.7)	20
Linda is a bank teller and is very shy. (T ∧ S)	**15.2** (**2.8**)	10
Linda is a teacher in elementary school and is active in crafts like needlepoint. (P ∧ C)	**31.4** (**3.6**)	30
T ∧ S interpreted as an intersection.^c^ (*n* = 25)	17.1 (3.7)	10
T ∧ S interpreted as two separations.^c^ (*n* = 11)	14.6 (6.4)	0
T ∧ S interpreted as neither an intersection nor two separations.^c^ (*n* = 4)	10.0 (5.4)	7.5

^a^In the version given to participants, the labels *P*, *F*, *T*, *Y*, *R*, *S*, *M*, *C*, *D*, *T* ∧ *F*, *Y* ∧ *P*, *D* ∧ *M*, *R* ∧ *F*, *T* ∧ *S* and *P* ∧ *C* were omitted

^b^Standard errors with 95 % confidence intervals are in parentheses. *Boldface* indicates a significant difference, relative to the conjunctions and their corresponding unlikely constituents (*p* < .05)

^c^Based on respondents’ choices in the Venn diagram task. Respondents were regarded as providing *an intersection*, *a disjunction*, or *neither an intersection nor a disjunction* interpretation when they chose respectively Option C, B, or any other option except for Option C and B in Fig. 2. There are so many more participants in Group 1 because Experiment 2 was conducted firstly through Group 1, however, the likelihood types of the Group 1’s statements are mostly the likelihood type of “Unlikely ∧ Likely” and have not enough data in relation to the types of “Likely ∧ Likely” and “Unlikely ∧ Unlikely”. On the other hand, some studies indicate that the conjunction fallacies are related to the likelihood types (e.g., Fantino et al. 1997; Nilsson et al. 2009; Tversky and Kahneman 1983; Yates and Carlson 1986) and that more conjunction fallacies should be happened in the likelihood type of “Unlikely ∧ Likely” rather than the likelihood types of “Unlikely ∧ Unlikely” and “Likely ∧ Likely” (e.g., Fisk 1996; Yates and Carlson 1986). Thereof, in order to examine the second prediction of the current paper, “Likely ∧ Likely” and “Unlikely ∧ Unlikely” combinations of likelihood types of the statements through latter three Groups are thereafter included. Needed numbers of the latter three Groups’ participants are employed to generate much more needed likelihood types

#### Materials

The questionnaires contained two pages and consisted of the following two parts:

The first part located at the first page and presented a modified Linda problem derived from Tversky and Kahneman (1983). For Group 1 to 4, after reading the same personality description of Linda (target *E*), participants in each group were instructed to estimate on probabilities of two conjunctive statements, their respective constituents (see Table 3 for the respective stimulus used in each group), and another irrelevant single statement. The order of the statements was counterbalanced. For each group, the independent variable was the two conjunctive statements and their constituents. Probability judgment and measures derived from these, such as conjunction fallacy, were dependent variables. The participants were invited to use a number in the whole range 0 to 1 (including 0 and 1) either by decimal, fractional, or percent estimates as a reply to each statement. It was mentioned in the instructions that when they forecast that a statement is *unlikely* to happen, they should determine its probability within the range of 0 to 0.50, and when they forecast that a statement is *likely* to happen, they should determine its probability within the range of 0.5 to 1, where 0 means minimal probability and 1 means maximal probability.

**Fig. 2:**
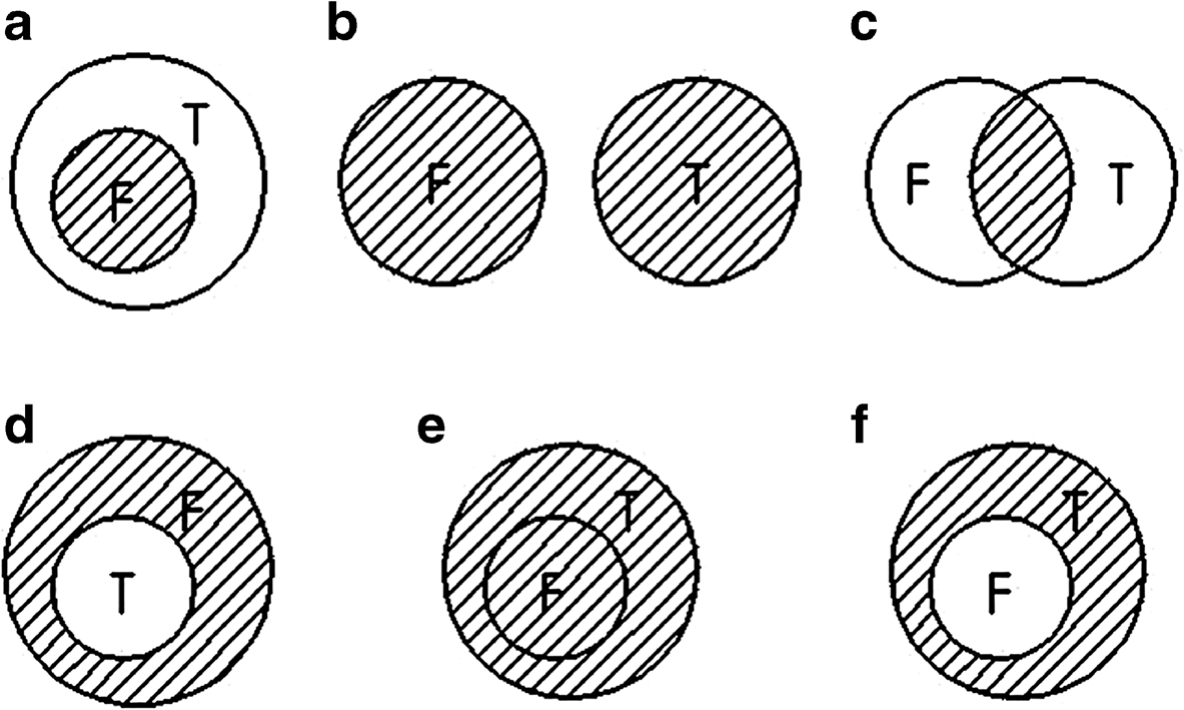
The Venn diagram task. *Note* the character “*T*” was replaced as “*R*” for Group 3 and the character “*F*” was replaced as “*S*” for Group 4

The second part located at the second page and presented a Venn diagram problem (see Fig. 2). The independent variable was the options of the Venn diagram. Choices and measures derived from these were dependent variables. By presenting the participants with six Venn diagrams options, the participants were asked to choose only one graphic option in which the part of shadow can describe best of their understanding on the conjunctive statement *T* ∧ *F* for Group 1 and 2, *R* ∧ *F* for Group 3, or *T* ∧ *S* for Group 4 according to their judgments in the first part task. Among the six diagrams options, the options (B) and (C) represent a disjunction of two statements and a conjunction of two statements, respectively. Therefore, this Venn diagram task may better understand whether participants comprehended the conjunction word “*and*” as a conjunction, disjunction interpretation, or another part of speech.

#### Procedure

The experiment was conducted in four quiet classrooms. As the study presented no more than minimal risk of harm to the participants and involved no procedures for which written consent is normally required outside of the study context, the Ethics Committee specially approved the study as well as verbal informed consent being provided to the participants in lieu of signed informed consent. The experimenter (myself) read out loud the verbal informed consent at the outset of the experiment. All of the participants consented to participate in the experiment. After that, the experimenter (myself) read out loud a general instruction to inform the participants that the questionnaires were designed to look for the way that people make their judgments under uncertain situation. Then the experimenter gave each participant one leaflet presented with instructions and questions. The participants were instructed to complete the first page’s probability judgments task before looking over the following page. They were also told not to go back to the first page and not to adjust their earlier probability judgments when answering the tasks in the second page. Questions were answered individually at the participants’ seats. Some participants were mixed together to complete the questionnaires through some groups.

### Results and Discussion

#### Probabilities

Participants whose answers for the first part were incomplete (by omitting probability judgments of at least one statement except for the unrelated statement in each group) were excluded from the analysis (*N* = 26). For the remaining 224 participants, the average and median probability estimates from Group 1 to 4 for the corresponding two conjunctive statements and their respective constituents are shown in Table 3, with standard errors (95 % confidence interval) in parentheses. Results of paired *t* test showed that the mean estimates for all of the conjunctions through Group 1 to 4 were significantly greater than the mean estimates for their corresponding unlikely constituents (*t* (103) = −7.723, *p* < .05 for *T* ∧ *F* and its corresponding unlikely constituent in Group 1; *t* (103) = −4.094, *p* < .05 for *Y* ∧ *P* and its corresponding unlikely constituent in Group 1; *t* (36) = −1.881, *p* < .05 for *T* ∧ *F* and its corresponding unlikely constituent in Group 2; *t* (36) = −3.724, *p* < .05 for *D* ∧ *M* and its corresponding unlikely constituent in Group 2; *t* (40) = −1.275, *p* < .05 for *R* ∧ *F* and its corresponding unlikely constituent in Group 3; *t* (40) = −1.925, *p* < .05 for *D* ∧ *M* and its corresponding unlikely constituent in Group 3; *t* (41) = −2.566, *p* < .05 for *T* ∧ *S* and its corresponding unlikely constituent in Group 4; *t* (41) = −3.066, *p* < .05 for *P* ∧ *C* and its corresponding unlikely constituent in Group 4), as shown in bold on significant effects.

#### Components and Conjunction Fallacy

A weighted average of 49.78 % single conjunction fallacy (min 34.2 %, max 64.5 %) and a weighted average of 16.7 % double conjunction fallacy (min 7.1 %, max 31.7 %) were made. As predicted, Chi-squared tests reveal that the single conjunction fallacy is higher than both of the zero conjunction fallacy (*χ*^2^ = 7.321, *p* < .01) and the double conjunction fallacy (*χ*^2^ = 37.752, *p* < .001).

To determine whether the results of the conjunction fallacy are exceptionally frequent for the likelihood type of the Unlikely-Likely combination of components, the likelihood types of the conjunctive statements’ constituents are classified as either *unlikely* (probabilities in the interval between 0 and 50 %, where 0 % included and 50 % excluded); *likely* (probabilities in the interval between 50 and 100 %, where 50 % excluded and 100 % included); or *no difference* (probabilities are equal to 50 %). All the conjunctive statements’ likelihood types (2 per participant) are classified into four groups: *Unlikely* ∧ *Unlikely*, *Likely* ∧ *Unlikely*, *Likely* ∧ *Likely*, and *intermediate*. Because the intermediate type of the conjunctive statements entails no difference, those data are excluded from the analyses that follow.

For the 199 responses classified as the Likely ∧ Unlikely type, 147 (73.9 %) responses committed conjunction fallacies. For the 166 responses classified as the Likely ∧ Likely or Unlikely ∧ Unlikely type, 97 (58.4 %) responses committed conjunction fallacies. Therefore, as predicted by the equate-to-differentiate model (Li 2004) and also as observed in previous studies (e.g., Fantino et al. 1997; Nilsson et al. 2009; Wedell and Moro 2008), more conjunction fallacy was observed when participants estimated the conjunctions including one likely and one unlikely components, as compared with when the conjunctions include either two likely or two unlikely components (*χ*^2^ = 9.730, *p* < .01).

The signed sum model (Yates and Carlson 1986) predicts that zero conjunction fallacy is most common in the Unlikely ∧ Unlikely type as compared to the Likely ∧ Likely and Likely ∧ Unlikely types, single conjunction fallacy is more frequent in the Likely ∧ Unlikely type rather than the Likely ∧ Likely and Unlikely ∧ Unlikely types, and double conjunction fallacy is numerous only in the Likely ∧ Likely type.

**Fig. 3:**
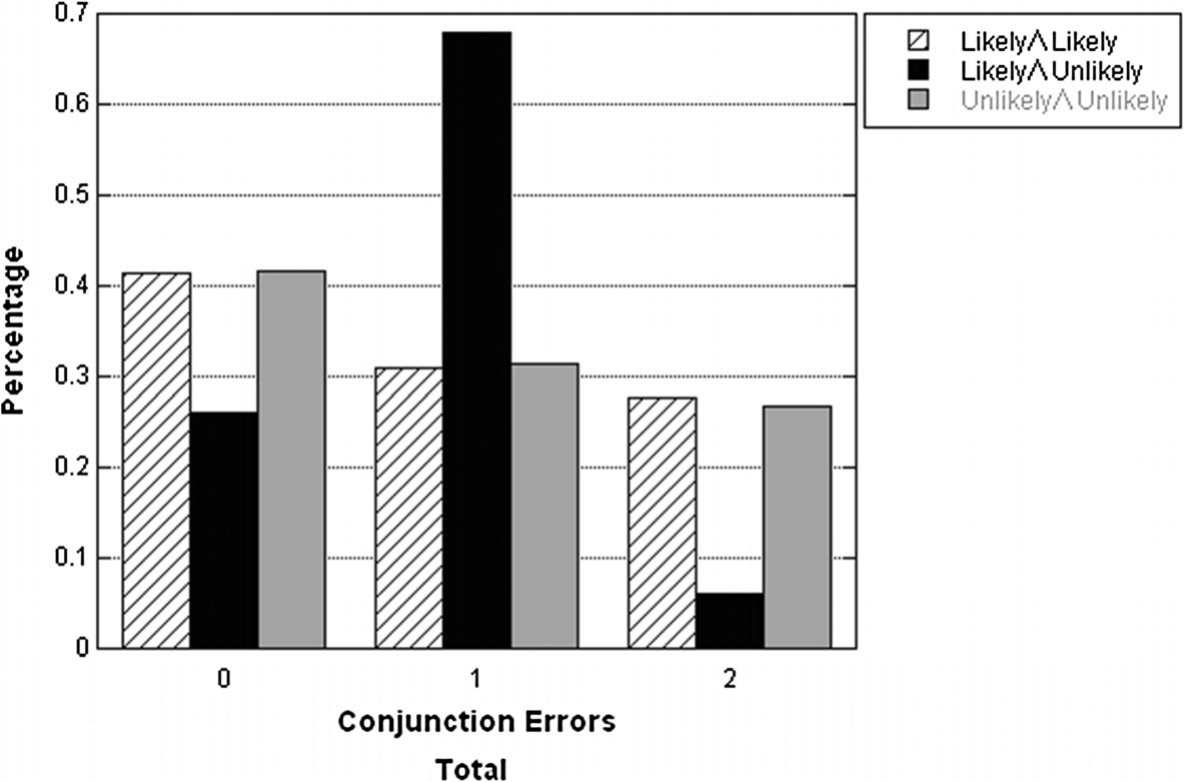
Percentages of zero, single, and double conjunction fallacies in Experiment 2 by combinations of component probability likelihood classification Likely ∧ Likely, Likely ∧ Unlikely, and Unlikely ∧ Unlikely

Figure 3 displays percentages of the participants in total who made zero, single, or double conjunction fallacy by the classification of the likelihood types Likely ∧ Likely, Likely ∧ Unlikely, and Unlikely ∧ Unlikely. Zero conjunction fallacy is most common in the type of Unlikely ∧ Unlikely as compared to the type of Likely ∧ Unlikely (*χ*^2^ = 7.811, *p* < .01), but not frequently different between the types of Unlikely ∧ Unlikely and Likely ∧ Likely (*χ*^2^ = 0.001, *p* = .971). Single conjunction fallacy is more frequent in the type of Likely ∧ Unlikely rather than the types of Likely ∧ Likely and Unlikely ∧ Unlikely (*χ*^2^ = 48.299, *p* < .001). Double conjunction fallacy is most common in the type of Likely ∧ Likely as compared to the type of Likely ∧ Unlikely (*χ*^2^ = 21.496, *p* < .001), but not frequently different between the types of Likely ∧ Likely and Unlikely ∧ Unlikely (*χ*^2^ = 0.010, *p* = .919). In sum, as also demonstrated previously by Fisk (1996) and in accordance with the results of Fisk and Pidgeon (1996), differences of the three likelihood types on zero and double conjunction fallacies are not in accordance with the signed sum model of qualitative likelihood judgment (Yates and Carlson 1986), but only the type of Likely ∧ Unlikely on single conjunction fallacy is in accordance with the signed sum model of qualitative likelihood judgment (Yates and Carlson 1986).

According to the CWA (Nilsson et al. 2009), P (*A* ∧ *B*) = *β*P (*A*) + (1-*β*) P (*B*) (where P (*A*) < P (*B*) and the coefficient *β* is the relative weight of the two components, .5 < *β* < 1). The CWA predicts that both P (*A*) and P (*B*) should influence P (*A* ∧ *B*), and that P (*A*) should attract more weight than P (*B*) if regression analyses are run with smaller and larger constituent probabilities as factors and conjunctive probabilities as dependent variables (also see Fisk 2002 and Cobos et al. 2003).

Multiple regression and partial correlation analyses (see Table 4) are run for the participants’ probability estimates of the respective six conjunctive statements, the total six conjunctive statements, and their corresponding smaller and larger components. The regression equation is $\overset{\frown }{P\left(A\wedge B\right)}$ = *c*_2_ + *β*_Large_ × P (*B*) + *β*_Small_ × P (*A*), where P (*A*) < P (*B*) and *c*_2_ is a constant term. Results of regression and partial correlation analyses were broadly consistent with the assertion of both the CWA (Nilsson et al. 2009) and the potential surprise (Fisk 2002) that the probability of the smaller component is invariably the more significant one in determining the probability of its conjunctive statement. As those participants in Group 3 approximately estimated *R* (mean = 72.1 %) and *F* (mean = 72.2 %) equally, neither the smaller nor larger component exerts significant influence on its conjunctive statement *R* ∧ *F*.

**Table 4:** Probability estimates of the larger and smaller component in determining the value assigned to the conjunctive statements in Experiment 2: regression and partial correlation analyses results

	*R* ^2^	Standard coefficient – *β*		Partial correlation coefficient – *β*		*N*
Statement		L*arge*	S*mall*	L*arge*	S*mall*	
T ∧ F	.120	.191^*^	.270^**^	.198^*^	.275^**^	141
Y ∧ P	.254	.222^*^	.377^***^	.232^*^	.376^***^	104
D ∧ M	.376	.250^*^	.449^***^	.264^*^	.442^***^	78
R ∧ F	.373	.376	.304	.332	.280	41
T ∧ S	.340	.296	.453^**^	.338	.481^**^	42
P ∧ C	.479	.174	.595^***^	.210	.591^***^	42
Total	.427	.278^***^	.486^***^	.316^***^	.503^***^	450

^*^ *p* < .05

^**^ *p* < .01

^***^ *p* < .001

#### Quantitative Predictions

To explore to what extent the equate-to-differentiate model provides a good quantitative account of the data, the participants’ probability estimates on the two conjunctive statements and their four components were studied. For a conjunctive statement, the outcomes of the less or most distinct dimension can be regarded as the probabilities of the corresponding component statements constituting to the conjunctive statement. For example, the probability of *T* can be regarded as the outcome of the less distinct dimension $\mathbb{T}$ of the conjunctive statement *T* ∧ *F*. The relative discrepancies between each pair of the outcomes of the conjunctive statements are assumed to determine whether the corresponding two dimensions are the less or most distinct dimensions. For example, since 45 of 104 participants in Group 1 judged the relative discrepancy between the probabilities of *F* and *P* as the most distinct one, the dimensions $\mathbb{F}$ and ℙ were assumed as the most distinct dimensions. Therefore, the less distinct dimensions $\mathbb{T}$ and $\mathbb{Y}$ were filtered, and the preference between *T* ∧ *F* and *Y* ∧ *P* was determined by the preference between *F* and *P*. Since they judged *F* ≻ _L_*P* and (*T* ∧ *F*) ≻ _L_ (*Y* ∧ *P*), their responses were consistent with the criterion of the equate-to-differentiate model.

In sum, 152 (67.9 %) of the 224 participants’ responses were in accordance with the predictions of the equate-to-differentiate model. Thus, the results were approximately consistent with the model’s account of the conjunctive probability judgment.

#### Venn Diagram Task

**Fig. 4:**
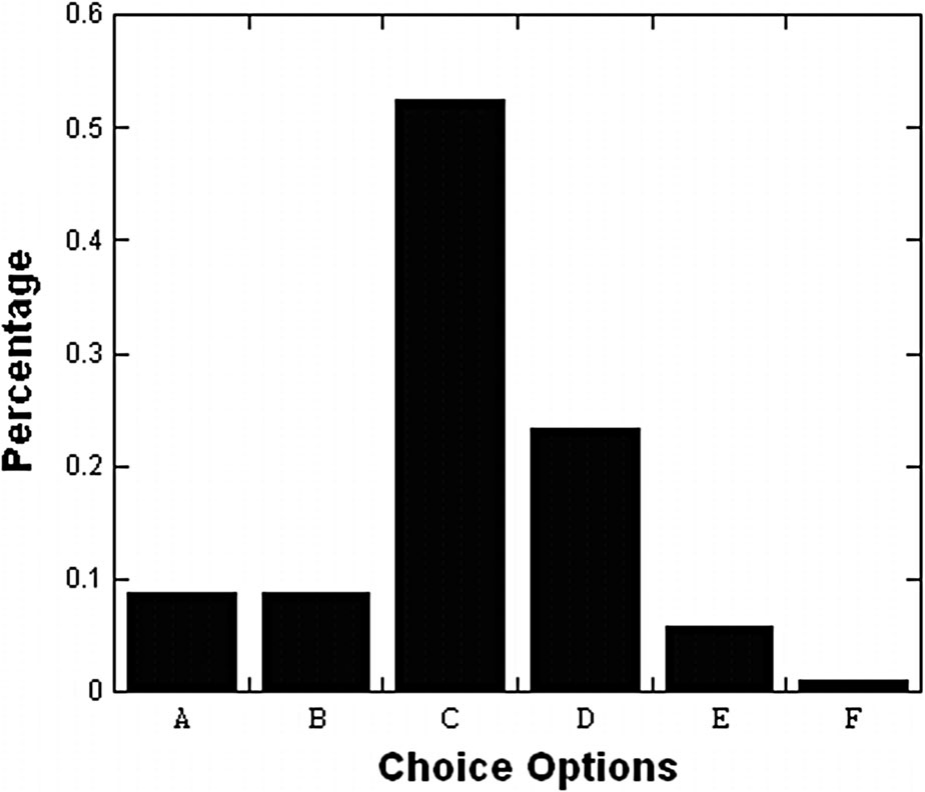
Results of the Venn diagram choices in Experiment 2. *Note* *N* = 218. Option B denotes *a disjunction* interpretation and option C denotes *an intersection* interpretation. See Fig. 2 for graphic representations of each option

Table 3 also shows the participants’ intersection, disjunction, and other interpretations on conjunctive word “*and*”, as inferred from results of the Venn diagram task shown in Fig. 4. Among the 224 participants, six participants were excluded from the Venn diagram task because two of them chose two options and the other four participants left empty answers. For the remaining 218 participants, 126 (57.8 %) and 32 (14.7 %) participants chose the intersection and disjunction interpretations respectively. Remaining 60 (27.5 %) participants chose neither the intersection nor disjunction interpretation.

For 126 (57.8 %) of the 218 participants who chose the intersection interpretation (Option C), only 28 (22.2 %) of the 126 participants were in accordance with the conjunction rule on their estimates of the corresponding two compound statements and their respective constituents. However, 27 (21.4 %) of the 126 participants estimated one of the compound statements and its constituents according to the conjunction rule, but violated the axiom on the other compound statement and its constituents. Still there were 71 (56.3 %) of the 126 participants who committed conjunction fallacies on the corresponding two compound statements and their respective constituents.

For 32 (14.7 %) of the 218 participants who chose the disjunction interpretation (Option B), only 2 (6.3 %) of the 32 participants were totally in accordance with their interpretation by estimating the four compound statements more probable than their corresponding two conjunctive statements. However, 6 (18.8 %) of the 32 participants were partly in accordance with their interpretation by committing single conjunction fallacies on the two compound statements and their respective constituents. Another 13 (40.6 %) of the 32 participants were partly in accordance with their interpretation by committed single or double conjunction fallacies on one of the compound statements and its constituents, but they did not violate the axiom on the other compound statement and its constituents. Conversely, even 11 (34.4 %) of the 32 participants were against their misinterpretation by estimating according to the conjunction rule on the two compound statements and their respective constituents. On the other hand, the 32 participants’ estimates on the conjunctive statements (mean = 26.1 %) were significantly different to the average estimate for their corresponding unlikely constituents (mean = 21.2 %, R^2^ = .616, correlation coefficient *ρ* = .745, and *p* < .001) and were not significant different to the average estimate for their corresponding likely constituents (mean = 60.5 %, R^2^ = .616, correlation coefficient *ρ* = .081, and *p* = .407). Those respondents’ misinterpretation of the conjunctions as the disjunctions seemingly does not lead them to depend on the larger components rather than the smaller ones to estimate the probabilities of the conjunctions.

For 32 (14.7 %) of the 218 participants who chose Option D, a representation of, for instance in Group 1, ¬ *T* ∧ *F* (Linda is not a bank teller and she is a feminist), by Bayes’ theorem,

1 $$ \begin{array}{l} Prob\left(\neg T\wedge F\right) = Prob\left(\neg T\right)\times Prob(F)\hfill \\ {}\kern5.9em  = \left[1\ \hbox{--} Prob(T)\right]\times Prob(F),\hfill \end{array} $$

on the assumption that *T* and *F* are independent.

Let

2 $$ t= Prob(T) $$

3 $$ f= Prob(F). $$

Then by substituting (2) and (3) into (1),

4 $$ Prob\left(\neg T\wedge F\right) = \left(1 - t\right) \times f. $$

Therefore when the following conditions are satisfied

5 $$ t < \left(1 - t\right) \times f $$

and *T* and *F* are independent,

the probability of ¬ *T* ∧ *F* is larger than *T*. That implies

6 $$ Prob(T) < Prob\left(\neg T\wedge F\right). $$

It indicated that when people misinterpret the conjunction *T* ∧ *F* as ¬ *T* ∧ *F* on the assumption that *T* and *F* are independent as well as that the conditional inequality (5) is satisfied, the probability of “*T* ∧ *F*” can be greater than the probability of its component *T*. Of those 32 participants who chose Option D, probability judgments of 27 (84.4 %) participants were satisfied with the conditional inequality (5). In this case, it indicated that the misinterpretation of the conjunctive statement as the conjunction of its base statement’s complement and its added statement may be a part of people’s errors in the conjunction fallacy. This finding echoes Politzer and Noveck’s (1991) argument that actually the conjunction fallacy is not fallacious in the circumstance when participants misinterpret a base statement as the conjunction of the base statement and the complement of an added statement.

## Conclusions

We live in a fundamentally uncertain world in which people with limited cognitive resources are regularly challenged to make correct judgment and decisions. How can people judge accurately, and moreover what are the mechanisms that authentically reflect people’s cognitive processes? Recent trends inspired from simple heuristics and ecological rationality suggest that people’s judgment processes are based on pragmatic heuristics and are a deductive modeling of bounded rationality as an alternative basis for achieving an optimal goal of a rational decision (see Gigerenzer and Goldstein 1996 for a discussion). Given the information available, pragmatic heuristics no longer view judgment as a fully rational process of optimization. Instead, heuristics assume that people apply their “rational” judgments only after the information is simplified appropriately because people either lack abilities or resources or are constrained by time pressure to attain the rational solutions. For example, Kahneman and Tversky (1973) found that people’s predictions do not adequately take into account the overall probabilities (or base rates) of statements. Therefore, conditional probability judgments of rare statements are often inflated. As a result, as people pursue satisfactory solutions rather than rational optimality, they usually “neglect” extra information by using pragmatic heuristics rather than by using relatively complicated calculations.

Resting on the pragmatic heuristics assumption set out above, the equate-to-differentiate model (Li 2004) was proposed in this paper to explain the conjunction and disjunction fallacies. The fundamental reasoning process of the model is not established upon traditional principles of rational theories (i.e., expected utility calculations), but arising from people’s ecological cognitive processing and combinations of simple considerations. The model assumes that mechanism of people’s judgment under risk and uncertainty is not to seek for a certain kind of expected value maximization, but to compare attributes of each statement rather than considering the integrate statement (accumulated evidence, e.g., Wang and Li 2012, in regard of comparison between statements found that attribute-based transitions are observed more frequently than statement-based transitions). By using the model, information of statements’ less distinct dimension(s) is screened and omitted first, and then the most important ones are reserved as judgment of the statements. Although omitting partial dimension(s) of statements leads to the wastage of partial information, this pragmatic process can be thought even better describing human reason pattern. Especially, the interpretation of the specific pragmatic algorithms seemingly leads to deficiency of Bayes’ probability judgment on conjunctive and component statements in the Linda problem as Gigerenzer and Goldstein (1996) have already demonstrated. It is conjectured that the conjunction and disjunction fallacies can be best reflected by a pragmatic intelligence as reasoning conjoined with the assumption of ecological thinking processes.

The aim in this paper is not to argue that the conjunction and disjunction fallacies can be explained by a rational, Bayes’ Rule-based account, though some studies have tried to give reasonable explanations on the fallacies according to probability theories (e. g., Costello 2009). This paper does not also prove that the fallacies can be explained by the part of experiential-intuitive or so-called automatic-heuristic system’s process in human reasoning (see dual-processing propositions, e.g., De Neys 2006; Epstein et al., 1996; Lu 2015, for a recent review; Stanovich and West, 1998). On the contrary, taking example by the viewpoints of ecological and bounded rationality, this paper, although preliminary, argues that under a complex reasoning situation (such as incorrect logic deduction, limited cognitive capacities, absence of knowledge, or lack of time), people intend to use a range of simple psychological mechanisms so-called the pragmatic heuristics to help them cope better with daily judgment and decision making (see Mosconi and Macchi 2001 for proposing a role of pragmatic rules in the conjunction fallacy).

The two rational and pragmatic camps are still debating whether the famous conjunction and disjunction fallacies are the consequence of people’s automatic intuitive thinking responses or the consequence of particular cognitive algorithms that people implement. Although thinking styles can be considered as a cognitive trait with stabile specialities and can be measurable with designated self-report, it is still difficult to distinguish when computative analytic processing or instinct experiential processing (e.g., the equate-to-differentiate model) is used in people’s reasoning. The class of pragmatic heuristics based on ecological and bounded rationality is possibly the actual result of an attempt to regulate the inner psychological balance between the emotional (intuitive) and the cognitive (rational) thinking modes.

The equate-to-differentiate model’s interpretation of the conjunction and disjunction fallacies has some distinct or close implications comparing with other alternative explanations introduced in *1.1 The Conjunction and Disjunction Fallacies*. First, models of the psychological relations (e.g., the representativeness and availability heuristics, Tversky and Kahneman 1983) are criticized as of vagueness and lack of theoretical specification. While the interpretation of the equate-to-differentiate model has its concrete judgment processes and theoretical assumptions (see *1.3 The Present Study*). Second, the misunderstanding hypothesis attributes to communication processes by assuming that people misinterpret connection relationship between statements or even the statements themselves by, for examples, misunderstanding ∧ as ∨, *T* | *E* as *E* | *T*, *T* ∧ *F* as *T* ∧ ¬ *F*, or *T* ∧ *F* as ¬*T* ∧ *F*. Similarly, the equate-to-differentiate model attributes to an individual mind (heuristics) by assuming that people follow a pragmatic, simple heuristic when estimating conjunction and its components. Furthermore, the equate-to-differentiate model assumes, similarly as misunderstanding ∧ as ∨, that the disjunctive connection (∨) or conjunctive connection (∧) is unvalued, and the comparison of the valued dimensions of each statement is regarded. Yet, in many cases, the expected behavior is similar, as it is for the data presented here as well (e.g., some participants understood the conjunction disjunctively on the Venn diagram). Third, several heuristic rules’ explanations, e.g., the conjunction coefficient model (Abelson et al. 1987) and the signed sum model of qualitative likelihood judgment (Yates and Carlson 1986), propose some relatively complicated computing processes so that those rules are hardly believed to reflect closely to people’s real implicit processes of judgments. In contrast, it is different from those complicated assumptions in that the equate-to-differentiate model’s interpretation put forward to a pragmatic judgment process that can be thought as reflecting closely to a real implicit process in people’s simple judgment’s needs. Compared with explanations from psychological relations (e.g., Tversky and Kahneman 1983) to misunderstanding (e.g., Hertwig et al 2008) to integrate computing models (e.g., Nilsson et al. 2009), the equate-to-differentiate model’s explanation is a cognitive adaptation. In cases of limited knowledge and computing ability, it is perhaps an ecological strategy a person can follow to exploit patterns of information in the environment to make judgments in a valid way.

In conclusion, the present paper proposes the equate-to-differentiate model (Li 2004) to explain the conjunction and disjunction fallacies. In two experiments, participants’ likelihood judgments of combinatorial and component statements were tested against the criterion of the model, and 82.9 and 67.9 % of the participants’ responses respectively in the disjunction fallacy and the conjunction fallacy were consistent with the prediction. The model was also compared against other three prevailing explanations of the phenomena, the CWA (Nilsson et al. 2009), the potential surprise model (Fisk 2002), and the signed sum model (Yates and Carlson 1986). The pattern of responses in Experiment 2 was not completely consistent with the signed sum model (Yates and Carlson 1986). The results presented in both Experiment 1 and 2 were broadly consistent with the CWA (Nilsson et al 2009) and the potential surprise (Fisk 2002) that the larger component probability assumes the key role in determining the disjunctive probability estimates while for conjunctions it is the smaller component (although the three models are based on different mechanism). Furthermore, the participants’ understood meaning of the semantic word “*and*” was studied by using a Venn diagram task to give insights on the misinterpretation hypothesis. Although over half participants (57.8 %) in total in Experiment 2 correctly interpreted the conjunctive statement as an intersection, 77.7 % of those participants still committed conjunction fallacy at least on one of the conjunctive statement and its component statements. For the participants who misinterpreted the conjunctive statement as a disjunction, 93.7 % of those participants were actually contrary to this misinterpretation by estimating at least one of the component statements more probable than its conjunctive statement. It indicates that participants’ judgments on the conjunction and its components are usually opposed to their interpretations on the connective word “*and*”, which gives rise to a disadvantage to the misinterpretation hypothesis. At the same time, the results in Experiment 2 also showed that the misinterpretation hypothesis cannot explain the conjunction fallacy in the case when participants misinterpret the conjunctive statement as a disjunction. However, the misinterpretation hypothesis may be correct when participants proper misinterpret the conjunctive statement as the conjunction of its base statement’s complement and its added statement.

## Footnotes

1 The notation “≽_L_” rather than “≽” is used to emphasize that the decision maker’s judgment may or may not satisfy all the probability axioms. The notation “≽” indicates that the axioms are satisfied according to probability theory’s definition.
